# Febrile Hashimoto's encephalopathy associated with Graves’ disease and acute pancytopenia: A case report

**DOI:** 10.1097/MD.0000000000018012

**Published:** 2019-12-16

**Authors:** Shaoyu Liu, Qingbian Ma, Yaan Zheng

**Affiliations:** From Department of Emergency Medicine, Peking University Third Hospital, Beijing, China.

**Keywords:** fever, Graves’ disease, Hashimoto's encephalopathy, neuropsychological symptom

## Abstract

**Rationale:**

Hashimoto's encephalopathy (HE) is an autoimmune-mediated encephalopathy rarely seen in Graves’ disease, with <20 cases reported previously, associated with elevated concentration of circulating serum anti-thyroid antibodies usually responsive to steroid therapy.

**Patient concerns:**

We present a HE case (25-year-old male) with Graves’ disease, complicated by fever and pancytopenia. The patient presented with fever, gait impairment, delirium, agitation and disorientation.

**Diagnoses:**

Thyroid-related antibodies were elevated and brain magnetic resonance imaging confirmed symmetrical white-matter lesion. There was no evidence of infection or other reasons to explain all of his clinical manifestations. Hashimoto's encephalopathy (HE) is an autoimmune encephalopathy with various manifestations and the characteristic of elevated anti-thyroid antibodies and has no relationship to thyroid function.

**Interventions:**

The patient had nonspecific clinical manifestations and excellently respond to glucocorticoid therapy.

The symptoms and the radiographic abnormalities disappeared after glucocorticoid therapy.

**Outcomes:**

We followed up with him for 5 years, in which there was no recurrence and his thyroid function continued to be normal.

**Lessons:**

It is important to evaluate thyroid function and related antibodies in patients present with neuropsychological symptoms to avoid delay in diagnosis.

## 1 Introduction

Hashimoto's encephalopathy (HE), also known as encephalopathy associated with autoimmune thyroid disease, is diagnosed as encephalopathy with an elevated concentration of circulating serum anti-thyroid antibodies, including antithyroglobulin antibody and antithyroid peroxidase antibody (anti-TPOAb, which is a composition of antithyroid microsomal antibody [anti-TMAb]).^[1]^ The HE diagnosis is based on exclusive criteria of no evidence of infection or other well-defined cerebral disorders.^[2,3]^

Almost all HE patients present with Hashimoto's thyroiditis; only a remarkably limited number of cases have been found associated with Graves’ disease. Moreover, the clinical manifestations of HE are extremely varied.

Here, we present a case of HE associated with Graves’ disease. The patient manifested fever and pancytopenia, and went into rapid remission after steroid therapy. Informed consent was given by the patient.

## 2 Case report

A 25-year-old Chinese man presented at the emergency department of Peking University Third Hospital in September 2013 because of fever and gait impairment. He had been initially diagnosed with Graves’ disease and dysthyroid ophthalmopathy 2 months before in another hospital with no previous medical condition, and was prescribed methimazole (30 mg/day). Leucopoenia was found 1 month before his appearance at our hospital, but he did not seek further treatment, and his leukocyte count was not monitored regularly.

The patient presented to our hospital with a fever lasting 2 days without known causes. His maximum body temperature had been 40°C, accompanied by sore throat, chills, fatigue, and diarrhoea. No other symptoms were reported. He had an acute onset of ataxia without losing consciousness 1 day before his appearance, and he developed agitation and disorientation soon after his arrival.

On initial assessment, the patient's body temperature was 38°C. His heart rate was 136 beats per minute. He was alert and exhibited delirium, dysarthria, and dyscalculia, but experienced no hallucinations. The patient also had a diffuse goitre without nodules or bruit. He was unable to perform finger-nose-finger tests or heel-to-shin test steadily, but muscle strength and tension of extremities were normal. There were no abnormalities in cranial and sensory nerve function. Meningeal and pathological signs were negative. The physical examination was unremarkable with regard to heart and lungs.

The brain computerized tomography (CT) was unremarkable, and blood analysis was performed immediately in the emergency room. Unsurprisingly, pancytopenia was found: white blood cell count was 0.29 × 10^9^ cells/L, hemoglobin 105 g/L, platelets 15 × 10^9^ cells/L, and neutrophils 0.02 × 10^9^ cells/L. Enteric infection was suspected based on fever and diarrhoea. Stool test and culture was negative; chest x-ray and urine test were both clear. Lumbar puncture was not performed because of thrombocytopenia and high risk of bleeding. There was no noticeable evidence of infection, but infection of the central nervous system could not be completely ruled out at the beginning. A blood culture was repeated as soon as possible, which yielded a negative result 7 days later.

In the meantime, a bone marrow cytology and biopsy were performed due to the pancytopenia and demonstrated myeloproliferative reduction (granulocyte count 1.5% and erythroid 3.5%). Results indicated no suspicions of leukemia or myeloproliferative disease. Thus, the pancytopenia was regarded as drug-related, based on the facts that it is the most common adverse effect of methimazole, and that it occurred after the prescription and before the onset of fever and mental disorder. An extensive blood workup for systemic immunity had negative results, including antinuclear antibodies, anti-ds-DNA, anti-ENA spectrum, and anti-neutrophil cytoplasmic antibodies.

Hyperthyroidism and Graves’ disease were confirmed instantly through free triiodothyronine (FT3) 6.93pg/mL, free thyroxine (FT4) 2.51 ng/dL, and thyroid-stimulating hormone (TSH) <0.08 μIU/mL (Table 1). Considering the thyroid state of our patient, it did not seem pessimistic for his condition to be explained by thyroid storm. We used β-blocker instead of anti-thyroid drugs immediately. Nevertheless, his mental disorders continued to gradually deteriorate.

**Table 1:**
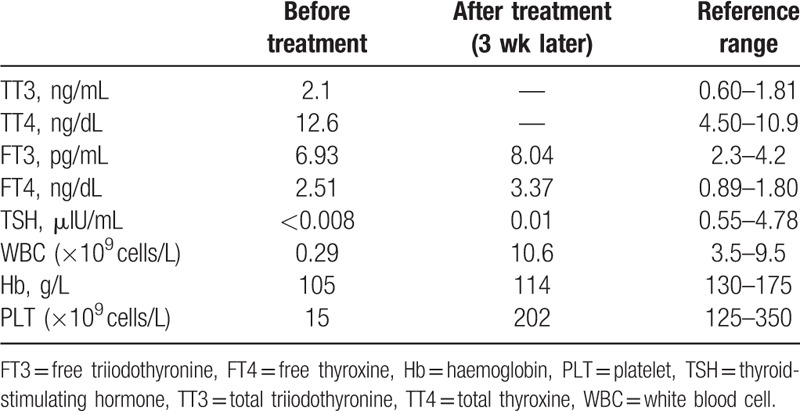
Laboratory data from hormone and common blood count during hospitalization.

**Figure 1:**
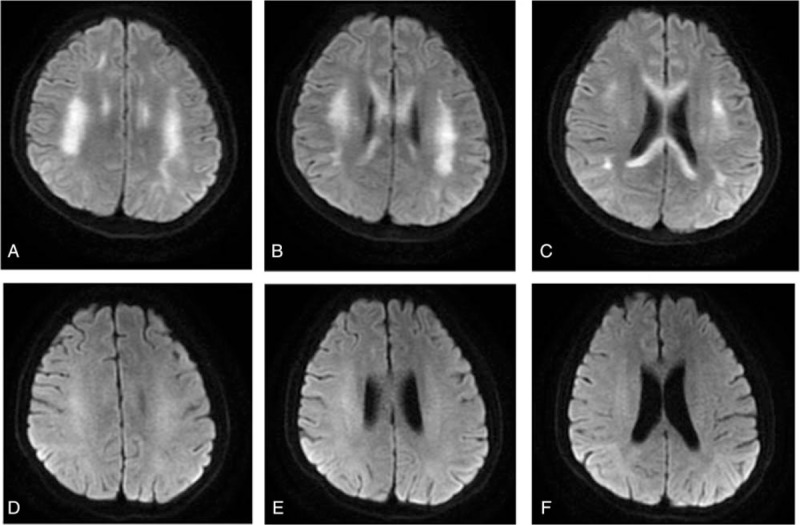
Brain magnetic resonance imaging before and after treatment. (A–C) High signal of white-matter (the corpus callosum, bilateral centrum semiovale, periventricular area) and symmetrical subcortical high signal lesions on diffusion-weighted imaging before treatment. (D–F) High signal lesions on the original MRI disappeared after treatment.

To further differentiate the origin of the neurological disorder, we performed a laboratory test for the unexpected but possible toxicosis. Then we conducted brain magnetic resonance (MR) imaging, which confirmed high signal white-matter (corpus callosum, bilateral centrum semi valve, periventricular area) and showed symmetrical subcortical high signal lesions on diffusion-weighted imaging and fluid-attenuated inversion recovery images (Fig. 1A–C). The study of anti-thyroid antibodies revealed elevated titers of TSH receptor autoantibodies (TRAb) (3.65U/L, normal<1.8 U/L) and anti-TMAb (>1300 U/mL, normal <60 U/mL). An electroencephalogram was not executed because the patient could not cooperate.

The patient was given meropenem (1 g intravenous infusion every 8 hours) starting immediately after arrival and continuing for 6 days, on suspicion of systemic infection with extremely low white blood cell count, before the final diagnosis was made. After leukemia was ruled out, granulocyte colony stimulation factor and recombinant human interleukin-11 were additionally given to the patient for agranulocytosis and thrombocytopenia. However, no obvious effect was observed. HE was suspected after a series of tests and imaging, as well as the patient's poor response to initial treatment. The patient was then given a high dose of glucocorticoid IV methylprednisolone (500 mg/day) for 3 days; from the fourth to the sixth day it was tapered to 240 mg/day, and then reduced by half to 120 mg/day for another 3 days. This regimen was followed by oral prednisolone (60 mg/day) as maintenance therapy.

On the next day after IV glucocorticoid therapy was initiated, the body temperature of the patient normalized and the delirium disappeared. On the fourth day of corticosteroid therapy, blood cell counts improved significantly (WBC: 2.39 × 10^9^ cells/L, neutrophils: 1.4 × 10^9^ cells/L, platelets: 30 × 10^9^ cells/L) and were back to normal at last by discharge (Table 1). On the fifth day, the signs and symptoms of ataxia disappeared. The cranial magnetic resonance imaging (MRI) was taken again on the 17th day, which showed that the subcortical high signal lesions on the original MRI had disappeared (Fig. 1D–F).

The patient underwent an uneventful hospital stay. He was put on thyroid radioiodine uptake test and radioiodine^131^ therapy before discharge. We followed up with him for 5 years, in which there was no recurrence and his thyroid function continued to be normal.

## 3 Discussion

HE is a rare disorder with an estimated prevalence of 2/100,000, most cases in adult females.^[1]^ Even though HE was first described by Brain et al in 1966,^[2]^ who indicated that it was associated with Hashimoto's thyroiditis, subsequent studies demonstrated that HE could also occur in patients with Graves’ disease. Such instances are extremely rare, with <20 cases ever reported in the world.^[4]^ Occasionally, previously reported and confirmed by pathology, Graves’ disease may also be associated with Hashimoto's thyroiditis, which might make the situation more complicated. Our patient was diagnosed at a young age with Graves’ disease and ophthalmopathy, with profoundly elevated thyroid hormone levels, and was treated with a sufficient dose of anti-thyroid drug for 2 months. However, his TMAb was positive, obviously in contrast to other thyroid antibodies, which might indicate comorbidity with Hashimoto's thyroiditis or Hashimotoxicosis. Unfortunately, we did not perform a needle biopsy on this patient. Thus, even though the thyroid function remains normal after 5 years, he still needs close follow-up monitoring to see whether hypothyroidism develops, so as to further confirm the final diagnosis of his primary thyroid disease.

Many studies have demonstrated that HE occurs in hyperthyroidism, euthyroidism, and even in subclinical thyroid dysfunctions.^[5]^ Chong et al^[3]^ reviewed 85 HE patients and found that the functional status of thyroid varied but the neurological symptoms were similar in all patients. The major reported clinical manifestations of HE have included 2 distinctive clinical subtypes, vasculitis and diffuse progressive. The patient either presented with stroke-like episodes, or relapsing courses of seizures (including status epilepticus and myoclonus) and temporary neurological deficits such as consciousness disturbance, dementia, tremor, and a variety of psychiatric presentations ranging from depression and mania to hallucinations and schizophrenia.^[1]^ Fever has also been reported.^[6]^ Viral meningitis or affective disorders being the primary suspicions, these nonspecific symptoms of HE may drive patients to the neurology or psychiatric clinic on their first visit and cause misdiagnosis or delay in diagnosis, neither of which are uncommon and may lead to serious consequences. For our patient, meningitis was unlikely due to his antibiotic treatment being ineffective and his dramatic response to steroid therapy. These responses supported the diagnosis of HE instead. In addition to infection, toxicity, and metabolic encephalopathy, thyroid storm might have been placed at the top of the list of differentiations due to his fever, tachycardia, and altered mental status. Yet the patient had been put on standard treatment for 2 months. The preexisting thyroid hormone was likely to have been exhausted, and his FT4 on arrival was only mildly elevated, suggesting that there was little chance of either severe thyrotoxicosis or thyroid storm. His acute encephalopathy onset and dramatic responsiveness to steroids without anti-thyroid treatment was more suggestive of HE. Brain CT and MRI results can be negative in some patients, but positive findings have included unspecific focal or diffuse hyperintensities of the subcortical white matter or cerebellum ischemic lesions. It has been reported in one case that the subcortical hyperintensities disappeared simultaneously with clinical improvement,^[7]^ as was the case with our patient.

Fortunately, HE may develop regardless of the primary disease and thyroid functional state.^[1]^ All patients with HE have an increase in the serum concentration of anti-thyroid antibodies; this increase is the characteristic marker of HE. Some of the literature has shown that the CSF anti-thyroid antibody may be more sensitive than serum anti-TPOAb. More recently, anti-*N*-terminal of α-enolase, which has a high prevalence and sensitivity in patients with HE, has also been proposed as a diagnostic marker for HE.^[1]^ Variability and nonspecificity of symptoms make HE a diagnosis of exclusion of other common diseases. At this time, there is no consensus on the diagnostic criteria for HE.^[1]^

Although the pathogenesis of HE is still obscure, one explanation might be mitochondrial disorder.^[7]^ Yet the most frequently mentioned pathogenesis is the autoimmune mechanism, based on the elevated antibody background. In support of this view, biopsies from patients with HE reveal lymphocytic infiltration involving small arterioles and venules. Imperiale et al^[8]^ regarded HE as an immune inflammation mediated by CD8^+^ T cell, and Japanese researchers found that IgG_4_ concentration was elevated in serum and cerebrospinal fluid in HE patients.^[9]^ Our patient underwent sufficient antithyroid treatment but still developed HE, which suggested that HE was based on autoimmune background, independent of the thyroid functional state. Moreover, as we have seen in our patients, as well as in cases previously published in the literature, episodes of encephalopathy have been reported following anti-thyroid medication or radioiodine treatment. This suggests that rapid correction of the thyroid state might have been the initiating factor due to follicular cell destruction and the subsequent thyroid antigen dispersion or blood–brain barrier disruption.^[10]^

A more precise definition of HE may be steroid-responsive encephalopathy associated with autoimmune thyroiditis because of its excellent response to glucocorticoid therapy, which makes steroids the first-line treatment^[1]^ with a 96% response rate.^[3]^ At present, there is no definite consensus on the recommended duration of steroid therapy in HE. The doses and schedules are extremely variable. Our patient had a good outcome following the IV administration of 500 mg methylprednisolone. The symptoms and signs completely disappeared and the lesions evident in the MRI improved significantly. Other effective treatments include plasmapheresis and intravenous immunoglobulin, methotrexate, azathioprine, rituximab, mycophenolate, and levetiracetam, all of which could be choices if steroids were contraindicated, ineffective, or causing the development of adverse effects.^[1]^ Thyroidectomy has been reported as an alternative therapeutic approach, used on a patient who relapsed after immunosuppressive treatment and then who completely remitted after thyroid removal.^[10]^ Although some patients suffer relapse,^[7]^ including a fatal case reported,^[11]^ the overall prognosis of HE is favorable. Remission can even occur spontaneously in some patients without treatment.

## 4 Conclusion

HE is an autoimmune encephalopathy with the characteristic of elevated anti-thyroid antibodies, and is one of the important reversible causes of subacute encephalopathy. We have reviewed its rare association with Graves’ disease, its possible coexistence with Hashimoto's thyroiditis, and have emphasized that HE may mimic thyroid storm and meningitis. Diagnosis is often delayed due to lack of specificity in the clinical profile and the difficulty in obtaining robust confirmation for the diagnosis in a short time. It is important to evaluate thyroid function and related antibodies in patients who present with neuropsychological symptoms. Hashimoto's encephalopathy should be recognized as a rare extra-thyroid manifestation of Graves’ disease.

## Acknowledgments

The authors thank all faculties in resuscitation area of Adult Emergency and Department of Radiology, Endocrinology and Neurology, who had been involved in diagnosis and treatment of the patient. Special thanks to Dr. Shu Li M.D. for spending time and effort on revising the manuscript and precious opinion on English writing. Thanks to Dr. Ci Tian for following up the patient. All the persons mentioned above have given permission to be named in the acknowledgements section.

## Author contributions

**Conceptualization:** Shaoyu Liu, Qingbian Ma.

**Data curation:** Shaoyu Liu, Yaan Zheng.

**Resources:** Shaoyu Liu.

**Supervision:** Qingbian Ma, Yaan Zheng.

**Validation:** Qingbian Ma.

**Writing – original draft:** Shaoyu Liu.

**Writing – review & editing:** Shaoyu Liu, Qingbian Ma.
